# Highly efficient ZnO/Au Schottky barrier dye-sensitized solar cells: Role of gold nanoparticles on the charge-transfer process

**DOI:** 10.3762/bjnano.2.73

**Published:** 2011-10-13

**Authors:** Tanujjal Bora, Htet H Kyaw, Soumik Sarkar, Samir K Pal, Joydeep Dutta

**Affiliations:** 1 Center of Excellence in Nanotechnology, School of Engineering and Technology, Asian Institute of Technology, P. O. Box 4, Klong Luang, Pathumthani 12120, Thailand; 2 Unit for Nano Science and Technology, S. N. Bose National Centre for Basic Sciences, Sector - III, Block - JD, Salt Lake, Kolkata 700098, India

**Keywords:** dye-sensitized solar cell, gold nanoparticle, picosecond spectroscopy, Schottky barrier, zinc oxide nanorod

## Abstract

Zinc oxide (ZnO) nanorods decorated with gold (Au) nanoparticles have been synthesized and used to fabricate dye-sensitized solar cells (DSSC). The picosecond-resolved, time-correlated single-photon-count (TCSPC) spectroscopy technique was used to explore the charge-transfer mechanism in the ZnO/Au-nanocomposite DSSC. Due to the formation of the Schottky barrier at the ZnO/Au interface and the higher optical absorptions of the ZnO/Au photoelectrodes arising from the surface plasmon absorption of the Au nanoparticles, enhanced power-conversion efficiency (PCE) of 6.49% for small-area (0.1 cm^2^) ZnO/Au-nanocomposite DSSC was achieved compared to the 5.34% efficiency of the bare ZnO nanorod DSSC. The TCSPC studies revealed similar dynamics for the charge transfer from dye molecules to ZnO both in the presence and absence of Au nanoparticles. A slower fluorescence decay associated with the electron recombination process, observed in the presence of Au nanoparticles, confirmed the blocking of the electron transfer from ZnO back to the dye or electrolyte by the Schottky barrier formed at the ZnO/Au interface. For large area DSSC (1 cm^2^), ~130% enhancement in PCE (from 0.50% to 1.16%) was achieved after incorporation of the Au nanoparticles into the ZnO nanorods.

## Introduction

The dye-sensitized solar cell (DSSC), also known as the Grätzel’s cell, has been one of the most extensively studied types of solar cell in the last two decades due to its potential widespread application attributed to lower manufacturing costs [1–3]. Currently one of the major issues hindering the rapid commercialization of DSSCs is their lower conversion efficiency compared to conventional solar cells. The maximum conversion efficiency of any DSSC reported to date is about 11% [4]. Due to the presence of various material interfaces in a DSSC, the probability of recombination of the electrons is high at each interface. Law et al. [5] proposed the use of single crystalline zinc oxide (ZnO) nanowires instead of the widely used titanium oxide (TiO_2_) porous thin film to reduce the probability of electron recombination in the DSSC by providing a direct pathway for the electrons to diffuse into the photoelectrode.

Both, ZnO and TiO_2_, are wide-band-gap semiconductors with almost comparable band gap (3.37 eV and 3.2 eV, respectively). Due to the direct band gap, higher exciton energy (60 meV compared to 4 meV for TiO_2_) [6–7], higher electron mobility (200 cm^2^·V^−1^·s^−1^) [8] and its ease of synthesis [9], ZnO is widely employed in various optoelectronic device applications. But, in DSSCs, the recombination of the electrons at the semiconductor/electrolyte interface is still unavoidable. Various attempts to control the recombination at the semiconductor/electrolyte interface by passivating the electrolyte-exposed part of the metal oxide film by means of different additives in the electrolyte [10–13] or by using the core-shell structure of various metal oxides [14–18] have been reported.

In this work we discuss the use of Au nanoparticles in a DSSC based on ZnO nanorods to improve the device performance through better charge separation in the photoelectrodes. In this regards, several reports have been previously published [19–23] demonstrating the rapid charge transfer and improved charge separation upon the incorporation of Au nanoparticles in ZnO- or TiO_2_-based photoelectrodes ultimately leading to enhanced DSSC performance. But none of these reports clearly explain the exact nature of the charge-transfer mechanism in the presence of Au nanoparticles. In this present work, through the time-correlated single-photon-count (TCSPC) spectroscopic technique, we explore the charge-transfer process in a ZnO/Au-nanocomposite photoelectrode for DSSC application. From the spectroscopic studies, we demonstrate that the enhancement in DSSC performance in the presence of Au nanoparticles with the ZnO nanorods is primarily due to the formation of a Schottky barrier at the ZnO/Au interface and not due to the faster electron injection from dye to ZnO. The effect of Au nanoparticles on the generation of photocurrent in the ZnO/Au-nanocomposite-based DSSC was studied for small- (0.1 cm^2^) as well as large-area (1 cm^2^) solar cells and the results are compared to those of a ZnO-nanorod DSSC without gold nanoparticles.

## Results and Discussion

**Figure 1:**
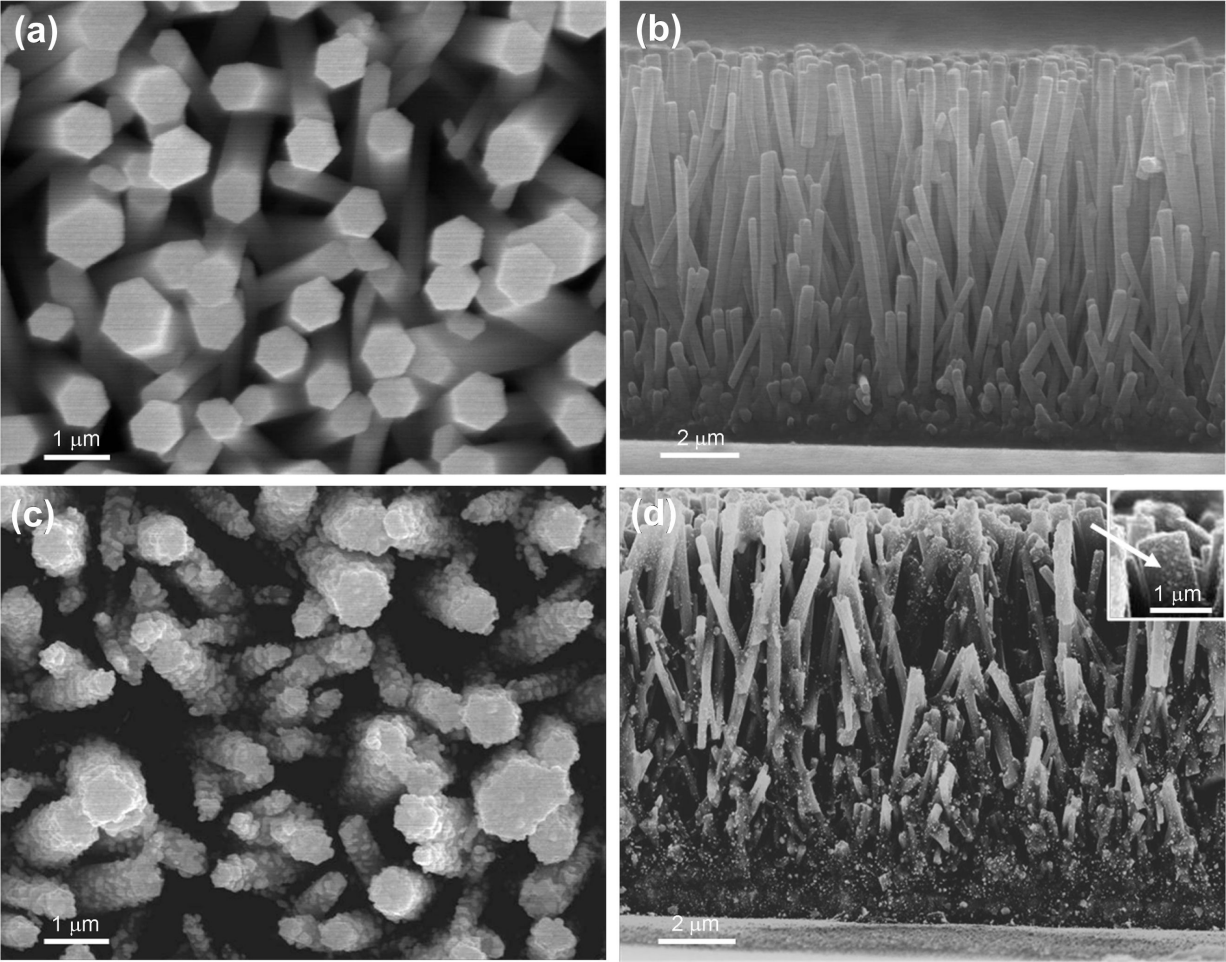
FESEM images showing (a) the top view and (b) the cross-sectional view of the bare ZnO-nanorod photoelectrode, and (c) the top view and (d) the cross-sectional view of the ZnO/Au-nanocomposite photoelectrode. The inset in (d) shows the magnified FESEM image of the Au nanoparticles as synthesized on the surface of the ZnO nanorods from a 0.01 mM HAuCl_4_·H_2_O aq. solution.

The surface morphology of hydrothermally grown ZnO nanorods and in-situ-deposited Au-nanoparticle-coated ZnO nanorods is shown in Figure 1, where the diameter of the ZnO nanorods was found to vary from ~500 to 700 nm and the length from ~8 to 9 μm. The distribution of the Au nanoparticles over the ZnO-nanorod surface was found to be fairly uniform, as can be observed from Figure 1c. The cross-sectional SEM image indicates (Figure 1d) that the Au nanoparticles grow almost homogeneously over the complete length of the ZnO nanorods.

**Figure 2:**
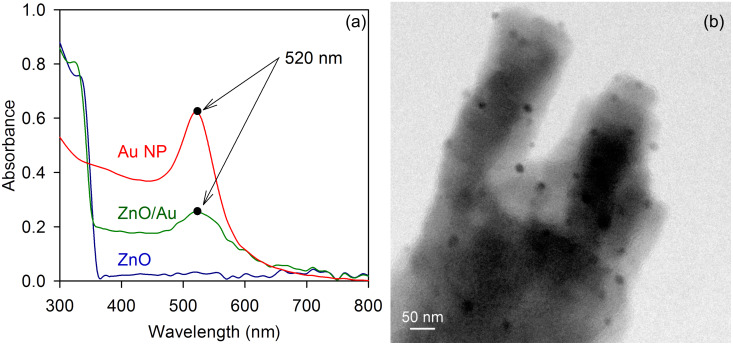
(a) Optical absorption of ZnO nanorods, ZnO/Au-nanocomposite photoelectrode and Au-nanoparticle colloid (particle size ~20 nm) and (b) TEM image of Au nanoparticles in situ deposited on the surface of a ZnO nanorod.

The optical absorptions of the ZnO-nanorod and ZnO/Au-nanocomposite photoelectrode are shown in Figure 2a. Due to the absorption by surface plasmons in the Au nanoparticles, a higher optical absorption of the ZnO/Au-nanocomposite photoelectrode near 520 nm was observed. The optical absorption by the ZnO/Au photoelectrode was compared with the absorption by Au nanoparticle colloids (particle size ~20 nm), and the results indicated that the Au nanoparticles in both systems were of comparable sizes, which was further verified by TEM imaging. A typical TEM image of the ZnO/Au-nanorod surface is shown in Figure 2b.

The solar cells with ZnO-nanorod and ZnO/Au-nanocomposite photoelectrodes in the absence of dye N719 were initially prepared in order to study the photovoltaic behavior of the ZnO/Au-nanocomposite system. The *J–V* characteristics of these solar cells are shown in Table 1. Upon illumination under a light intensity of 100 mW/cm^2^ (1 sun, air mass (AM) 1.5 G), the bare ZnO-nanorod solar cell exhibited short-circuit current density (*J*_sc_) of 18.80 μA/cm^2^ and open-circuit voltage (*V*_oc_) of 0.27 V. On the other hand, the ZnO/Au-nanocomposite solar cell, under illumination, demonstrated higher *J*_sc_ (82.46 μA/cm^2^) as well as *V*_oc_ (0.39 V) compared to the bare ZnO-nanorod solar cell, which is mainly attributed to the higher optical absorption of the ZnO/Au photoelectrode due to the absorption by surface plasmons in Au nanoparticles.

**Table 1:** *J–V* characteristics of bare ZnO-nanorod and ZnO/Au-nanocomposite solar cells in the absence of dye N719, measured at an illumination of 1 sun, AM 1.5 G (100 mW/cm^2^).^a^

*J–V* parameters	Bare ZnO-nanorod solar cell	ZnO/Au- nanocomposite solar cell

*V*_oc_ (V)	0.27	0.39
*J*_sc_ (µA/cm^2^)	18.80	82.46
fill factor, FF (%)	30.94	52.05
η (%)	0.002	0.017

^a^The active area of all the solar cells was maintained at 0.25 cm^2^ during these experiments.

In the case of the ZnO/Au solar cells without any sensitizer dye molecules, the photoexcited electrons in the Au nanoparticles are transferred to the conduction band (CB) of ZnO, and then diffuse through the ZnO nanorods towards the conducting fluorine-doped tin oxide (FTO) substrate resulting in higher photocurrent and photovoltage, as observed. The injection of the excited electrons from Au nanoparticles into the CB of ZnO is believed to be facilitated by the existence of the Schottky barrier at the ZnO/Au interface, which provides a unidirectional pathway for the electrons from gold to the conduction band of ZnO. Upon injection, the regeneration of the Au nanoparticles occurs by capturing electrons from the redox electrolyte (I^−^/I_3_^−^) present in the cell. Stability of the Au nanoparticles in the I^−^/I_3_^−^ redox electrolyte was tested by measuring the *J–V* characteristics of the ZnO/Au solar cell after 24 h (data not shown here), and negligible changes in the solar cell performance were observed.

**Figure 3:**
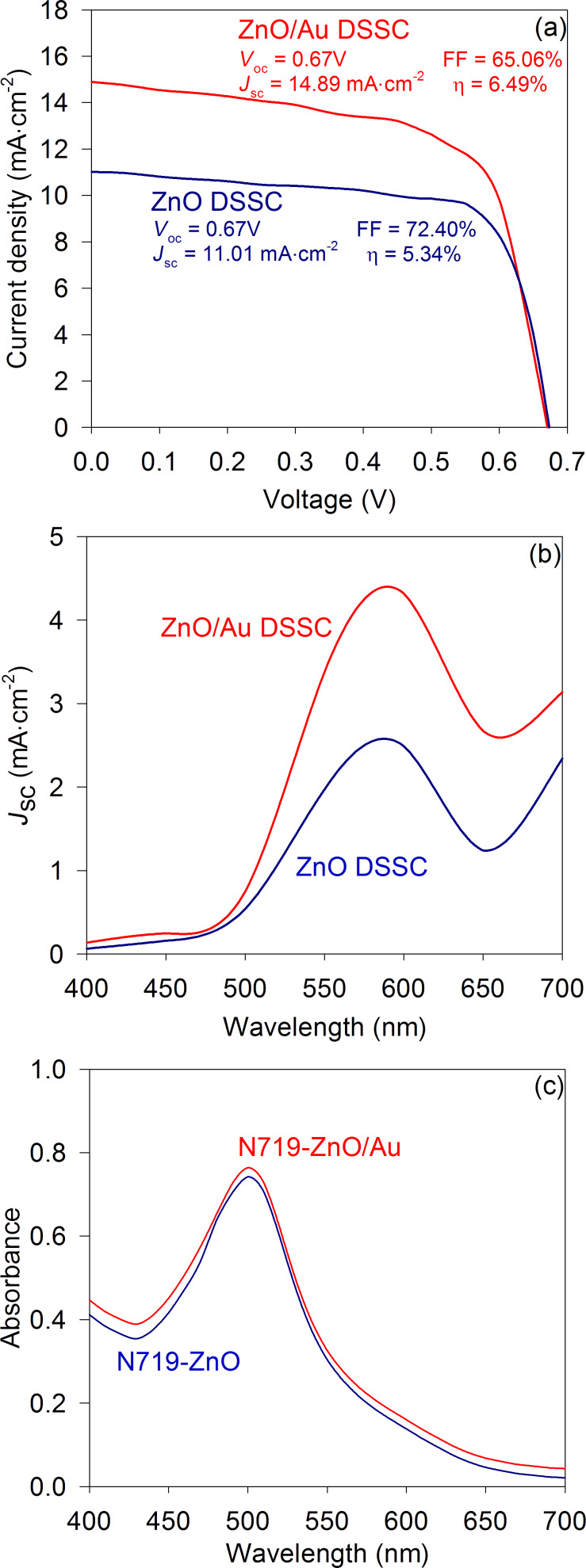
(a) *J–V* characteristics of the bare ZnO-nanorod and ZnO/Au-nanocomposite DSSCs, measured at 1 sun, AM 1.5 G illumination, (b) short-circuit photocurrent density of the bare ZnO-nanorod and ZnO/Au-nanocomposite DSSCs measured at different incident wavelengths and (c) optical absorptions of dye N719 in 0.1 mM KOH aqueous solution for bare ZnO-nanorod and ZnO/Au-nanocomposite photoelectrodes. The optical absorption was measured by removing the dye molecules from the respective photoelectrodes (size = 1 cm^2^) by dipping them in a 0.1 mM KOH aqueous solution (2 mL) for 5 min.

The performance of the solar cell in the presence of the dye was then studied by fabricating three sets of ZnO-nanorod and ZnO/Au-nanocomposite solar cells, each with dye N719, and their *J–V* characteristics were measured under 1 sun, AM 1.5 G illumination; the results are shown in Table 2. In Figure 3a, the best *J–V* characteristics obtained for both bare ZnO-nanorod and ZnO/Au-nanocomposite DSSCs are shown. It was found that the photocurrent of the ZnO-nanorod DSSC improved upon the incorporation of Au nanoparticles in the ZnO-nanorod photoelectrode. For the ZnO/Au-nanocomposite DSSC, ~35% improvement in *J*_sc_ (14.89 mA/cm^2^) was obtained compared to the bare ZnO-nanorod DSSC (11.01 mA/cm^2^) and the overall power-conversion efficiency improved from 5.34% to 6.49%.

**Table 2:** *J–V* characteristics of bare ZnO-nanorod and ZnO/Au-nanocomposite DSSCs measured at 1 sun, AM 1.5 G illumination (100 mW/cm^2^).^a^

*J–V* parameters	Bare ZnO-nanorod DSSC				ZnO/Au-nanocomposite DSSC		
		
	cell I	cell II	cell III		cell I	cell II	cell III

*V*_oc_ (V)	0.67	0.67	0.67		0.67	0.65	0.66
*J*_sc_ (mA/cm^2^)	10.98	11.01	10.84		14.89	14.28	14.45
FF (%)	71.55	72.40	70.31		65.06	63.00	65.78
η (%)	5.27	5.34	5.11		6.49	5.85	6.28

^a^The active area of all the DSSCs was maintained at 0.1 cm^2^ during these experiments.

In Figure 3b, the photocurrent responses of the bare ZnO-nanorod and ZnO/Au-nanocomposite DSSCs measured at different incident wavelengths are shown. Due to the absorption by surface plasmons in the Au nanoparticles, an improved photocurrent response was observed above 500 nm illumination in the case of the ZnO/Au-nanocomposite DSSC compared to the bare ZnO-nanorod DSSC. The ZnO/Au-nanocomposite photoelectrodes also showed slightly higher dye adsorption compared to the bare ZnO-nanorod photoelectrode, as shown in Figure 3c, due to the high surface area provided by the Au nanoparticles embedded in the surface of the ZnO nanorods. However, due to the only marginal improvement observed in this case, its effect on the photocurrent generation was negligible.

The improved device performance observed in the case of the ZnO/Au-nanocomposite DSSC can also be attributed to the presence of the Schottky barrier at the ZnO/Au interface, which blocks the back electron transfer from the CB of ZnO to the I^−^/I_3_^−^ redox electrolyte.

The formation of the Schottky barrier at the ZnO/Au interface can be explained from the classical Schottky model, according to which a Schottky barrier forms at a semiconductor/metal junction when the work function of the metal (θ_M_) is higher than the electron affinity of the semiconductor (χ_S_) and the barrier height (θ_SB_) at the junction can be expressed as given in Equation 1.

[1]
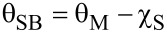


In the ZnO/Au-nanocomposite system, due to the larger work function of Au (5.1 eV) [21] compared the electron affinity of ZnO (4.2 eV) [24], a Schottky barrier exists at their interface. However, it has been well documented that gold can form both ohmic as well as Schottky junctions with n-type ZnO, depending on the crystal defects of ZnO [24–25]. In this regard, Brillson and coworkers [26–27] demonstrated that by removing the surface defects of ZnO, a ZnO/Au ohmic junction can be converted to a Schottky junction. Therefore, in the present study, in order to ensure the formation of a Schottky barrier at the ZnO/Au interface, the ZnO/Au photoelectrodes were annealed at 450 °C in air for 30 min after the deposition of the Au nanoparticles in order to remove the surface defects of ZnO.

**Figure 4:**
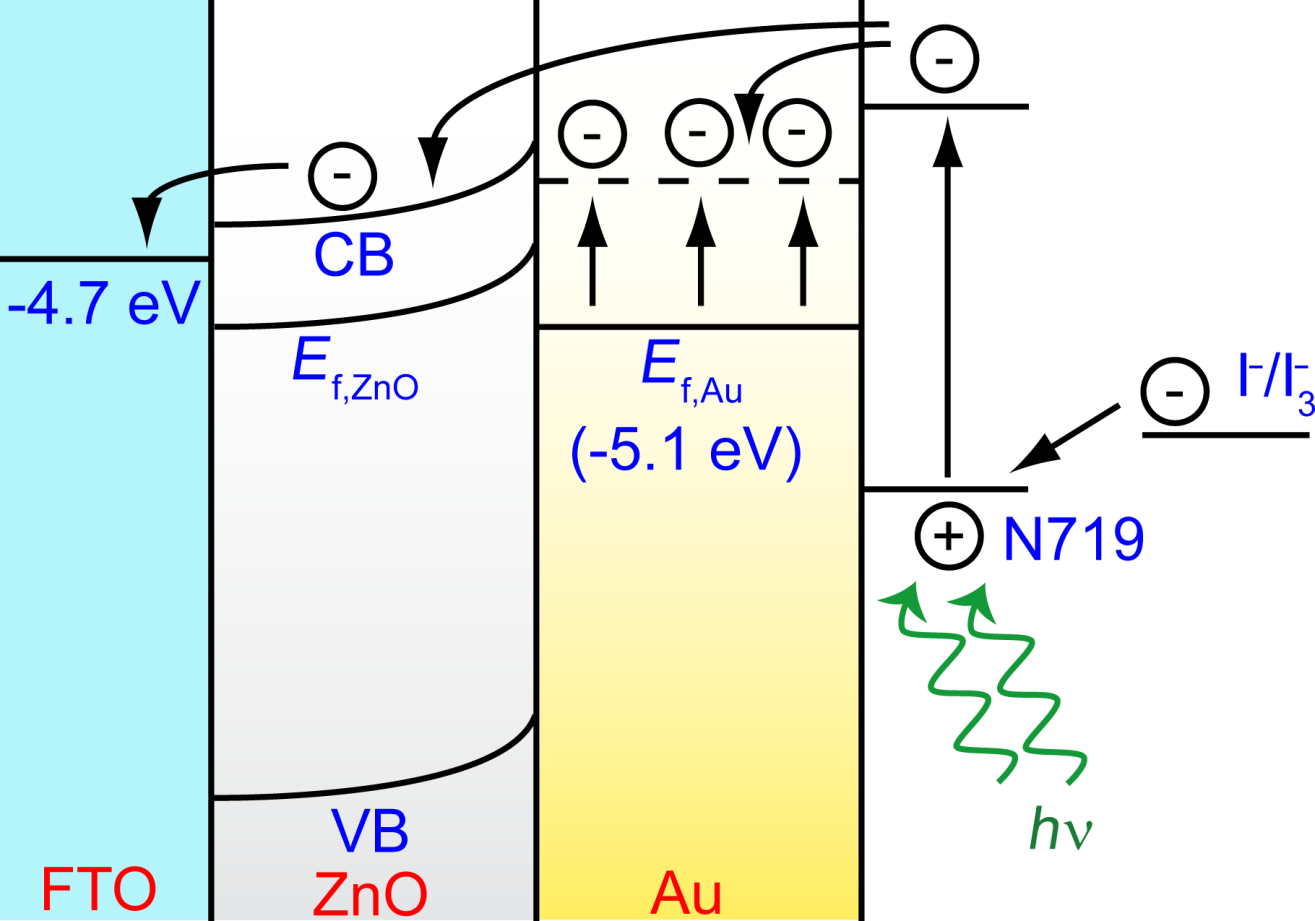
Energy-band diagram depicting the possible electron-transfer path in the ZnO/Au-nanocomposite DSSC, showing the Schottky barrier formed at the ZnO/Au interface. The dashed line in Au represents the position of the Fermi level of gold after electron injection from dye N719.

The formation of the Schottky barrier in the ZnO/Au-nanocomposite system and the possible electron-transfer path in the ZnO/Au DSSC is schematically represented in Figure 4. Upon irradiation, the electrons from excited dye molecules are injected into the Au nanoparticles embedded in the surface of the ZnO nanorods, resulting in an accumulation of electrons in the Au nanoparticle. As a result, the Fermi energy of the Au nanoparticles is pushed closer to the CB of the ZnO, and the transfer of electrons from the Au nanoparticles to the CB of ZnO could occur to establish charge equilibrium in the system. Some of the electrons from the dye can also be directly injected into the CB of the ZnO. In contrast, due to the existence of the Schottky barrier at the ZnO/Au interface, electrons at the CB of ZnO cannot reverse their path, and they flow towards the oxidized dye molecules or the redox electrolyte, thus leading to an improvement in the photocurrent.

**Figure 5:**
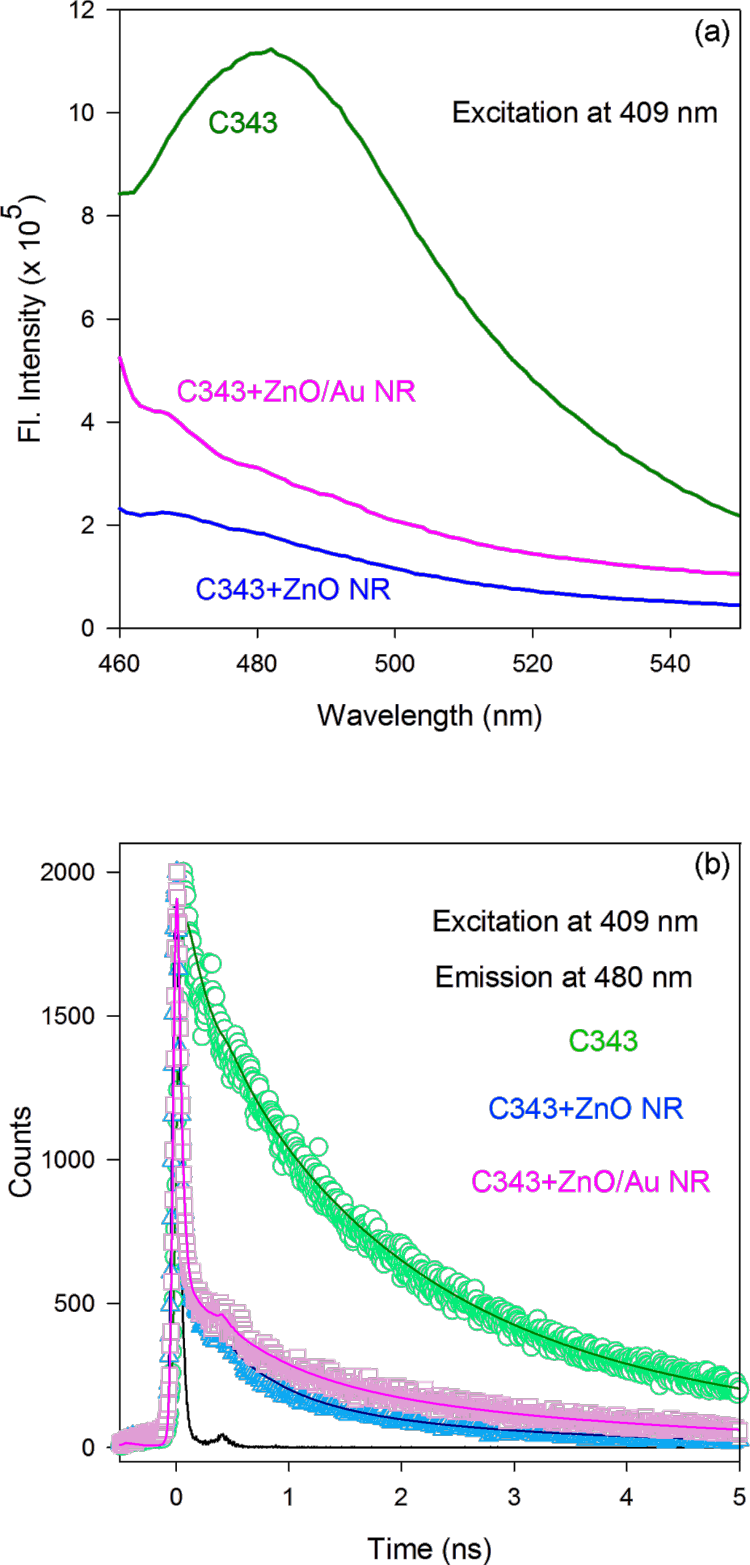
(a) PL spectra and (b) PL transients of the C343 dye observed at 480 nm in the presence and absence of bare ZnO nanorods and ZnO/Au nanocomposites. The IRF was 50 ps (FWHM) and all the samples were excited at a laser wavelength of 409 nm.

The charge-transfer mechanism in the ZnO/Au-nanocomposite system was elucidated by comparing the dynamics of the electron transfer from dye molecules to the bare ZnO and ZnO/Au-nanocomposite systems through TCSPC spectroscopy. For these experiments, dye N719 was replaced by a fluorescent dye, namely Coumarin 343 (C343). Similar to dye N719, the carboxylic group of C343 binds directly to the Zn atoms on the surface of the ZnO nanorods [28] and has been used in DSSC applications [29–30]. The steady-state photoluminescence (PL) spectra of the C343 dye, peaking at ~480 nm, in the absence and presence of both the bare ZnO-nanorod and ZnO/Au-nanocomposite systems is shown in Figure 5a, where the quenching of the PL intensity of C343 dye can be clearly observed in the presence of both ZnO nanorods and ZnO/Au nanocomposites. The PL transients measured at 480 nm (excitation at 409 nm) in the presence and absence of the ZnO-nanorod and ZnO/Au-nanocomposite systems are shown in Figure 5b. We observed a very sharp decay in the fluorescence intensity of the C343 dye in the presence of the ZnO-nanorod or ZnO/Au-nanocomposite system. This sharp decay (nonradiative path), observed here, indicates an efficient electron transfer from the sensitizer to the semiconductor system. The various decay time constants obtained after deconvolution of the fluorescence decay curves (Figure 5b) with the instrument response function (IRF) are given in Table 3.

**Table 3:** Dynamics of picosecond-resolved luminescence transients of C343 dye in the presence and absence of bare ZnO nanorods and ZnO/Au nanocomposites.^a^

Samples	τ_1_ (ns)	τ_2_ (ns)	τ_3_ (ns)	τ_avg_ (ns)

C343	3.866 (26%)	1.494 (48%)	0.191 (26%)	1.772
C343 + ZnO	2.508 (2%)	0.560 (4%)	0.015 (94%)	0.087
C343 + ZnO/Au	3.282 (3%)	0.691 (3%)	0.016 (94%)	0.134

^a^The emissions from C343 dye (probed at 480 nm) were detected with a 409 nm laser excitation. Numbers in the parentheses indicate the relative weighting.

The fraction of electrons following the fastest decay path (τ_3_ in Table 3) increased sharply from 26% to 94% in the presence of both ZnO-nanorod and ZnO/Au-nanocomposite systems, confirming the presumption that efficient electron transfer from the sensitizer dye molecules to the semiconductor system takes place. We have observed similar fluorescence-decay time constants (τ_3_) in both ZnO-nanorod and ZnO/Au-nanocomposite systems, indicating that the dynamics of the charge-transfer process from the surface-adsorbed sensitizer to the CB of the semiconductor is the same in both systems. In contrast, a very low electron population in the slow decay path (τ_1_ in Table 3) indicates that much less recombination of electrons occurs at the C343/semiconductor interface. The longer decay time constant observed in the case of the ZnO/Au-nanocomposite system (3.282 ns) compared to the bare ZnO-nanorod system (2.508 ns) clearly indicates a longer residence time of the electrons in the nanocomposite system before their recombination. This observed longer residence time can be attributed to the presence of the Schottky barrier in the ZnO/Au-nanocomposite system, which acts as a blocking layer for the electrons in the CB of ZnO and prevents them from recombining.

The performance of the ZnO/Au-nanocomposite DSSC was further studied for solar cells with larger active areas. The *J–V* characteristics for bare ZnO-nanorod and ZnO/Au-nanocomposite DSSCs with active areas of 0.25 cm^2^ and 1 cm^2^ are shown in Table 4. For an active area of 0.25 cm^2^, the addition of Au effectively increased the *J*_sc_ of the DSSC from 7.98 mA/cm^2^ for the bare ZnO-nanorod DSSC to 8.61 mA/cm^2^ for the ZnO/Au DSSC. The *V*_oc_ and fill factor (FF) was also increased from 0.63 V to 0.66 V and from 47.92% to 57.53%, respectively, indicating lower charge recombination at the photoelectrode due to the existence of the Schottky barrier in the ZnO/Au-nanocomposite DSSC as compared to the case of the bare ZnO-nanorod DSSC. As a result, the overall power-conversion efficiency improved from 2.41% to 3.27%.

**Table 4:** *J–V* characteristics of bare ZnO-nanorod and ZnO/Au-nanocomposite DSSCs with different active areas, measured at 1 sun, AM 1.5 G illumination (100 mW/cm^2^).

DSSC active area (cm^2^)	Bare ZnO-nanorod DSSC					ZnO/Au-nanocomposite DSSC			
		
	*V*_oc_ (V)	*J*_sc_ (mA/cm^2^)	FF (%)	η (%)		*V*_oc_ (V)	*J*_sc_ (mA/cm^2^)	FF (%)	η (%)

0.25	0.63	7.98	47.92	2.41		0.66	8.61	57.53	3.27
1.00	0.54	2.25	41.16	0.50		0.67	3.80	45.52	1.16

Similar results were observed in the case of ZnO-nanorod and ZnO/Au-nanocomposite DSSCs with an active area of 1 cm^2^. The overall power conversion efficiency (PCE) improved from 0.5% to 1.16% in the case of the ZnO/Au DSSC (*J*_sc_ = 3.80 mA/cm^2^, *V*_oc_ = 0.67 V and FF = 45.52%) compared to the bare ZnO-nanorod DSSC (*J*_sc_ = 2.25 mA/cm^2^, *V*_oc_ = 0.54 V and FF = 41.16%). The lower PCE observed in the case of the large-area DSSCs compared to the small-area DSSCs is mainly attributed to the increase in the sheet resistance of the conducting FTO substrates resulting in an overall increase in the series resistance (*R*_s_) of the large area DSSCs, which plays an important role in the performance of the solar cell [31–32]. Table 5 shows the series resistance (*R*_s_) of the ZnO/Au-nanocomposite DSSC for different active areas of the solar cells calculated by using a one-diode equivalent-circuit model for the DSSC as described by Murayama et al. [33–34]. It was observed that with increasing active area of the DSSCs from 0.1 cm^2^ to 1 cm^2^, *R*_s_ considerably increased from 5.13 Ω to 27.08 Ω, affecting the performance of the solar cells significantly.

**Table 5:** Series resistance (*R*_s_) of the ZnO/Au-nanocomposite DSSC measured for different active areas of the solar cells using a one-diode equivalent-circuit model for the DSSC.

ZnO/Au-DSSC active area (cm^2^)	Series resistance *R*_s_ (Ω)

0.10	5.13
0.25	10.10
1.00	27.08

The dependency of the device performance on the amount of Au nanoparticles in the photoelectrode was further studied by varying the dipping time of the ZnO-nanorod photoelectrodes in the 0.01 mM HAuCl_4_·H_2_O solution from 30 min to 2 h. The *J–V* characteristics of these ZnO/Au-nanocomposite DSSCs with an active area of 1 cm^2^ are shown in Table 6. On increasing the dipping time from 30 min to 1 h the overall performance of the ZnO/Au DSSC with increasing amount of Au nanoparticles in the photoelectrode was observed to increase. But for higher amounts of incorporated Au nanoparticles in the ZnO-nanorod photoelectrode (upon increasing the dipping time beyond 1 h) a drop in all the *J–V* parameters was observed: The *J*_sc_ was observed to decrease from 3.80 mA/cm^2^ to 2.40 mA/cm^2^, and *V*_oc_ and FF also dropped from 0.67 V and 45.52% to 0.62 V and 31.31%, respectively, when the dipping time was increased from 1 h to 2 h, resulting in a lower power-conversion efficiency, decreasing from 1.16% to 0.47%.

**Table 6:** *J–V* characteristics of ZnO/Au-nanocomposite DSSCs with increasing amount of Au nanoparticles in the photoelectrode as a function of the dipping time of ZnO-nanorod photoelectrodes in the HAuCl_4_·H_2_O aq. solution (0.01 mM).^a^

Dipping time (h)	*V*_oc_ (V)	*J*_sc_ (mA/cm^2^)	FF (%)	η (%)

0.50	0.64	3.10	41.58	0.83
1.00	0.67	3.80	45.52	1.16
1.50	0.65	2.90	34.33	0.65
2.00	0.62	2.40	31.31	0.47

^a^The *J–V* characteristics were measured at 1 sun, AM 1.5 G illumination (100 mW/cm^2^) and the active area of all the DSSCs was maintained at 1 cm^2^ during these experiments.

The reduction in the DSSC performances observed with a higher amount of Au nanoparticles in the photoelectrode is believed to arise due to the agglomeration of the Au nanoparticles on the ZnO-nanorod surface. With a smaller size of Au nanoparticles, the energy levels in Au are discrete and a greater shift in the Fermi levels is expected for a small electron accumulation in the Au nanoparticles [35–36]. But as Au nanoparticles agglomerate and form larger particles, more electron accumulation is required to achieve the upward shift of the Fermi level. As a result, most of the accumulated electrons from the larger Au nanoparticles recombine with the oxidized dye N719 molecules or with the I^−^/I_3_^−^ redox electrolyte before they can be transferred to the CB of ZnO, and thus this results in a poor photocurrent and low fill factor, as observed.

## Conclusion

Dye-sensitized solar cells with a ZnO/Au nanocomposite as the photoelectrode were successfully fabricated and their performances were compared with the bare ZnO-nanorod DSSC and discussed. Incorporation of Au nanoparticles into the ZnO-nanorod photoelectrode led to higher optical absorption by the photoelectrode and high dye intake, resulting in an ~35% enhancement in the photocurrent in the case of the ZnO/Au-nanocomposite DSSC (active area = 0.1 cm^2^) with *J*_sc_ equal to 14.89 mA/cm^2^ compared to the bare ZnO-nanorod DSSC with *J*_sc_ equal to 11.01 mA/cm^2^. As a result, the overall power-conversion efficiency was increased from 5.34% to 6.49% for the small-area (0.1 cm^2^) ZnO/Au-nanocomposite DSSC. Time-correlated single-photon count spectroscopic studies were conducted by using fluorescent Coumarin 343 dye, to explain the observed improvement in the DSSC performance in the presence of Au nanoparticles in the ZnO-nanorod photoelectrode. The results showed a longer decay time of the injected electrons at the CB of ZnO in presence of Au nanoparticles compared to the bare ZnO-nanorod samples, indicating a lower probability of electron recombination at the ZnO/Au interface resulting from the blocking of back electron transfer due to the existence of the Schottky barrier at this interface. A comparable decay time constant observed for the sharp fluorescence decay path associated with the injection of the electrons from the sensitizer dye to the CB of ZnO, in the presence and absence of Au nanoparticles, also revealed similar electron-transfer dynamics in both the bare ZnO-nanorod and ZnO/Au-nanocomposite systems irrespective of the presence of the Schottky barrier. In the case of the large area ZnO/Au DSSCs, efficiency dropped to 3.27% and 1.16% for active areas equal to 0.25 cm^2^ and 1 cm^2^, respectively, which was mainly attributed to the increased sheet resistance of the FTO substrates. For higher amounts of Au nanoparticles incorporated into the ZnO-nanorod photoelectrode, an ~60% reduction in the overall PCE of the ZnO/Au DSSC was observed, suggesting that the amount of Au nanoparticles in the ZnO-nanorod photoelectrode is crucial for optimizing the performance of the ZnO/Au-nanocomposite DSSC.

## Experimental

### 

#### Preparation of ZnO/Au-nanocomposite photoelectrode

All chemicals used in this study were analytical grade and were used without any further purification. Zinc acetate dihydrate (Zn(CH_3_COO)_2_·2H_2_O, Merck), zinc nitrate hexahydrate (Zn(NO_3_)_2_·6H_2_O, Sigma-Aldrich) and hexamethylenetetramine (C_6_H_12_N_4_, Aldrich) were used as starting materials for the growth of the ZnO nanorods. A low-temperature hydrothermal process was used for the ZnO-nanorod growth. Detailed processes for the hydrothermal growth of the single crystalline ZnO nanorods are described in our previous reports [9,37–39]. Briefly, a 1 mM zinc acetate solution in isopropanol was used to prepare the ZnO seed layer on a FTO substrate followed by annealing in air at 350 °C for 5 h. A 20 mM aqueous solution of zinc nitrate and hexamethylenetetramine was used as a precursor solution for ZnO-nanorod growth and the seeded FTO substrates were dipped into it at 90 °C for 40 h. As-grown ZnO nanorods on the FTO substrates were then taken out from the precursor solution and rinsed with DI water several times to remove unreacted residues from the substrate. Finally the substrates with ZnO nanorods were annealed at 350 °C for 1 h in air.

ZnO/Au-nanocomposite photoelectrodes were prepared by in situ precipitation of Au nanoparticles onto the surface of the ZnO nanorods. The process used to synthesize the Au nanoparticles on the ZnO-nanorod surface was slightly varied from the commonly followed process for the synthesis of Au nanoparticle colloids as described by Sugunan et al. [40]. A diluted 0.01 mM aqueous solution of gold chloride hydrate (HAuCl_4_·H_2_O, Aldrich) was prepared by adding 1 mL of 5 mM HAuCl_4_·H_2_O aqueous solution to 50 mL of DI water. The solution was then stirred for 15 min to mix the solute properly, and the photoelectrodes with ZnO nanorods were dipped into this diluted HAuCl_4_·H_2_O solution for 1 h. Separately, another diluted solution of 1.85 mM of trisodium citrate (TSC, C_6_H_5_Na_3_O_7_·2H_2_O, Merck) was prepared by adding 2 mL of 25 mM aqueous solution of TSC to 25 mL of DI water under continuous stirring for 15 min, and was used to precipitate gold nanoparticles. The photoelectrodes were then removed from the gold chloride solution and placed on a hot plate at 120 °C. Aqueous TSC solution (200 μL, 1.85 mM) was then dropped onto the photoelectrode in order to reduce gold chloride into gold nanoparticles, and the substrate was then allowed to dry. In order to reduce the unreacted gold chloride, TSC (200 μL) was added another couple of times followed by the drying sequence as explained above. Finally the photoelectrodes were cooled to room temperature and rinsed several times with DI water in order to remove any loosely attached gold nanoparticles from the ZnO nanorods. Subsequently the photoelectrodes were annealed at 450 °C for 30 min to ensure a good contact of the Au nanoparticles with the ZnO nanorods.

#### Fabrication of dye-sensitized solar cell

Bare ZnO-nanorod and ZnO/Au-nanocomposite photoelectrodes were then dipped into a 0.5 mM ethanolic solution of dye N719 and kept in the dark. Dye adsorption was carried out for 24 h, after which the photoelectrodes were removed from the dye solution and rinsed several times with ethanol in order to remove weakly adsorbed dye molecules. The reddish photoelectrodes were then kept in the dark for about 30 min and allowed to dry at room temperature. Platinized FTO glass was used as a counter electrode. A thin platinum layer was deposited on FTO-coated glass substrates by thermal decomposition of platinum chloride (H_2_PtCl_6_·H_2_O, Fluka) at 385 °C for 15 min. The counter electrode was then placed on top of the photoelectrode and a single layer of 50 μm thick surlyn 1702 (Dupont) was used as a spacer between the two electrodes. The DSSCs were then sealed and filled with the liquid electrolyte, consisting of 0.5 M lithium iodide (LiI), 0.05 M iodine (I_2_) and 0.5 M 4-*tert*-butylpyridine (TBP) in acetonitrile (ACN), by using capillary force, through two small holes (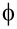 = 1 mm) drilled on the counter electrode. Finally the two holes were sealed by using another piece of surlyn to prevent the electrolyte from leaking out of the cell.

#### Sample preparation for the fluorescence study

For the fluorescence study, nanorod thin ﬁlms of ZnO and ZnO/Au were prepared on quartz glass substrate and the dye N719 was replaced with a fluorescent dye Coumarin 343 (C343, Aldrich), since N719 is nonfluorescent in nature. A 2 mM ethanolic solution of C343 dye was prepared and the nanorod thin ﬁlms were sensitized by immersing them in the C343 solution for1 h. After sensitization, the ﬁlms were washed with ethanol to remove weakly adsorbed dye molecules and dried under ambient conditions.

#### Characterization

The characterization of the ZnO nanorods and Au-nanoparticle-coated ZnO nanorods (ZnO/Au nanocomposite) was performed by means of scanning electron microscopy (SEM, JEOL JSM-6301F) and transmission electron microscopy (TEM, JEOL JEM-2010). Optical absorptions of the photoelectrodes were measured by using a UV–vis spectrophotometer from Ocean Optics (Micropack DH-2000) with USB4000 detector. Steady-state emission spectra were measured with a Jobin Yvon Fluoromax-3 fluorimeter (pump power at 320 nm is ~22 μW/cm^2^). Each of the photoluminescence transients was measured by the picosecond-resolved time-correlated single-photon counting (TCSPC) technique, with a commercially available picosecond diode laser-pumped (LifeSpec-ps) time-resolved fluorescence spectrophotometer from Edinburgh Instruments, U.K. Picosecond excitation pulses from the picoquant diode laser were used at 409 nm with an instrument response function (IRF) of 50 ps. A microchannel plate photomultiplier tube (MCP-PMT, Hammamatsu) was used to detect the photoluminescence from the sample after dispersion through a monochromator. For all transients, the polarizer on the emission side was adjusted to be at 55° (the “magic angle”) with respect to the polarization axis of the excitation beam. Measurements of the DSSC *J–V* characteristics were performed under 1 sun, AM 1.5 G (air mass 1.5 global, 100 mW/cm^2^) illumination by using a 150 W small-beam solar simulator (Sciencetech, model SF150) as a light source and a Keithley 617 programmable electrometer as a voltage source.

## References

[1] O'Regan B, Grätzel M (1991). Nature.

[2] Grätzel M (2004). J Photochem Photobiol, A.

[3] Grätzel M (2003). J Photochem Photobiol, C.

[4] Nazeeruddin M K, De Angelis F, Fantacci S, Selloni A, Viscardi G, Liska P, Ito S, Takeru B, Grätzel M (2005). J Am Chem Soc.

[5] Law M, Greene L E, Johnson J C, Saykally R, Yang P (2005). Nat Mater.

[6] Yang P, Yan H, Mao S, Russo R, Johnson J, Saykally R, Morris N, Pham J, He R, Choi H J (2002). Adv Funct Mater.

[7] Xia J B, Zhang X W (2006). Eur Phys J B.

[8] Quintana M, Edvinsson T, Hagfeldt A, Boschloo G (2007). J Phys Chem C.

[9] Baruah S, Dutta J (2009). Sci Technol Adv Mater.

[10] Nazeeruddin M K, Kay A, Rodicio I, Humphry-Baker R, Müller E, Liska P, Vlachopoulos N, Grätzel M (1993). J Am Chem Soc.

[11] Haque S A, Tachibana Y, Willis R L, Moser J E, Grätzel M, Klug D R, Durrant J R (2000). J Phys Chem B.

[12] Wang P, Zakeeruddin S M, Moser J E, Grätzel M (2003). J Phys Chem B.

[13] Gregg B A, Pichot F, Ferrere S, Fields C L (2001). J Phys Chem B.

[14] Palomares E, Clifford J N, Haque S A, Lutz T, Durrant J R (2003). J Am Chem Soc.

[15] Greene L E, Law M, Yuhas B D, Yang P (2007). J Phys Chem C.

[16] Wang M, Huang C, Cao Y, Yu Q, Deng Z, Liu Y, Huang Z, Huang J, Huang Q, Guo W (2009). J Phys D: Appl Phys.

[17] Zhang X T, Sutanto I, Taguchi T, Tokuhiro K, Meng Q B, Rao T N, Fujishima A, Watanabe H, Nakamori T, Uragami M (2003). Sol Energy Mater Sol Cells.

[18] Law M, Greene L E, Radenovic A, Kuykendall T, Liphardt J, Yang P (2006). J Phys Chem B.

[19] Barazzouk S, Hotchandani S (2004). J Appl Phys.

[20] Mikroyannidis J A, Stylianakis M M, Suresh P, Roy M S, Sharma G D (2009). Energy Environ Sci.

[21] Chen Z H, Tang Y B, Liu C P, Leung Y H, Yuan G D, Chen L M, Wang Y Q, Bello I, Zapien J A, Zhang W J (2009). J Phys Chem C.

[22] Chou C S, Yang R Y, Yeh C K, Lin Y J (2009). Powder Technol.

[23] Su Y H, Lai W H, Teoh L G, Hon M H, Huang J L (2007). Appl Phys A: Mater Sci Process.

[24] Brillson L J, Yicheng L (2011). J Appl Phys.

[25] Ip K, Thaler G T, Yang H, Han S Y, Li Y, Norton D P, Pearton S J, Jang S, Ren F (2006). J Cryst Growth.

[26] Brillson L J, Mosbacker H L, Hetzer M J, Strzhemechny Y, Look D C, Cantwell G, Zhang J, Song J J (2008). Appl Surf Sci.

[27] Brillson L J, Mosbacker H L, Hetzer M J, Strzhemechny Y, Jessen G H, Look D C, Cantwell G, Zhang J, Song J J (2007). Appl Phys Lett.

[28] Duncan W R, Prezhdo O V (2007). Annu Rev Phys Chem.

[29] Hara K, Sayama K, Ohga Y, Shinpo A, Suga S, Arakawa H (2001). Chem Commun.

[30] Zhang X, Zhang J J, Xia Y Y (2008). J Photochem Photobiol, A.

[31] Onoda K, Ngamsinlapasathian S, Fujieda T, Yoshikawa S (2007). Sol Energy Mater Sol Cells.

[32] Denhoff M W, Drolet N (2009). Sol Energy Mater Sol Cells.

[33] Murayama M, Mori T (2006). Thin Solid Films.

[34] Murayama M, Mori T (2008). Equivalent circuit analysis of dye-sensitized solar cell fabricated at low-temperature. Proceedings of the International Symposium on Electrical Insulating Materials ISEIM 2008.

[35] Kamat P V (2008). J Phys Chem C.

[36] Haruta M (1997). Catal Today.

[37] Baruah S, Dutta J (2009). J Sol-Gel Sci Technol.

[38] Baruah S, Dutta J (2009). J Cryst Growth.

[39] Sugunan A, Warad H C, Boman M, Dutta J (2006). J Sol-Gel Sci Technol.

[40] Sugunan A, Dutta J (2005). MRS Online Proceedings Library.

